# The patient-side surgeon plays a key role in facilitating robot-assisted intracorporeal ileal conduit urinary diversion in men

**DOI:** 10.1007/s11701-021-01256-x

**Published:** 2021-06-03

**Authors:** Yutaro Sasaki, Masayuki Takahashi, Kyotaro Fukuta, Keito Shiozaki, Kei Daizumoto, Keisuke Ozaki, Yoshiteru Ueno, Megumi Tsuda, Yoshito Kusuhara, Tomoya Fukawa, Yasuyo Yamamoto, Kunihisa Yamaguchi, Hirofumi Izaki, Kazuya Kanda, Hiroomi Kanayama

**Affiliations:** 1 Department of Urology, Tokushima University Graduate School of Biomedical Sciences, 3-18-15 Kuramoto, Tokushima, 770-8503 Japan; 2 Department of Urology, Tokushima Prefectural Central Hospital, 1-10-3 Kuramoto, Tokushima, 770-8539 Japan

**Keywords:** Patient-side surgeon, Surgical technique, Robot-assisted intracorporeal ileal conduit urinary diversion, Minimally invasive surgery

## Abstract

The influence of the console surgeon on the feasibility and outcome of various robot-assisted surgeries has been evaluated. These variables may be partially affected by the skills of the patient-side surgeon (PSS), but this has not been evaluated using objective data. This study aimed to describe the surgical techniques of the PSS in robot-assisted radical cystectomy (RARC) and intracorporeal ileal conduit (ICIC) urinary diversion and objectively examine the changes in surgical outcomes with increasing PSS experience. During a 3-year period, 28 men underwent RARC and ICIC urinary diversion. Clinical characteristics and surgical outcomes were compared between patients who underwent surgery early (first half group) or late in the study period (second half group). The pre-docking incision enabled easy specimen removal. The glove port technique widened the working space of the PSS. The stay suture allowed the PSS to control the distal portion of the conduit, facilitating the passage of the ureteral stents. During stoma creation, pneumoperitoneum pressure was lost by opening the abdominal cavity. To overcome this problem, the robotic arm was used to lift the abdominal wall to maintain the surgical field and facilitate the PSS procedure. Compared with the first half group, the second half group had significantly shorter times for urinary diversion (202 min vs 148 min, *p* < 0.001), ileal isolation and anastomosis (73 min vs 45 min, *p* < 0.001), and stenting (23.0 min vs 6.5 min, *p* < 0.001). As the experience of the PSS increased, the time of the PSS procedures decreased.

## Introduction

The literature on robot-assisted surgery focuses on the feasibility, surgical outcomes, and the experience or learning curve of the main console surgeon (CS) [1]. However, these variables may partially depend on the skills of the patient-side surgeons (PSS). In urologic surgery, the PSS plays a key role in robot-assisted radical cystectomy (RARC), especially in intracorporeal urinary diversion (ICUD); however, there is a lack of objective data to support this. The purpose of this study was to describe the surgical techniques of the PSS and to objectively examine the changes in surgical outcomes with increasing experience of the PSS.

The minimum requirements for the PSS are (1) to fully understand the surgical process and always act proactively so that the CS can concentrate on the surgery, (2) to learn to operate the forceps while minimizing interference with the robot arm and robot forceps, and (3) to understand how to use various instruments, such as electrosurgical scalpels, vessel sealing devices, ultrasonic coagulation and incision devices, ligation clips, and automated anastomotic devices. In addition, the PSS must promote the smooth performance of ICUD and aim for minimally invasive surgery (MIS). Among the surgical processes in ICUD, the most important surgical steps for the PSS to contribute to the achievement of a smooth operation while maintaining MIS are the creation of the ileal conduit, removal of the specimen, passage of the ureteral stent, and creation of the stoma. Herein, we describe the surgical techniques used by the PSS to create the intracorporeal ileal conduit (ICIC) and objectively examine the surgical outcomes.

## Materials and methods

For this retrospective cohort study, we reviewed the medical records of the patients with bladder cancer who underwent RARC in Tokushima University Hospital and Tokushima Prefectural Central Hospital from January 2018 to December 2020. During the study period, 92 patients underwent RARC. Of these, 39 had undergone ICIC urinary diversion. The specimens were removed transvaginally from women, and so women were excluded from this study. The final study cohort comprised 28 men who underwent RARC and ICIC urinary diversion. The institutional review board approved this study (protocol number 3838), and all patients provided informed consent. All surgeries were performed by eight CS and were assisted by one PSS (Y.S.). RARC was indicated for patients with muscle invasive bladder cancer (T2–T4a, N0–Nx, M0) and high risk and recurrent non-muscle-invasive bladder cancer, as well as extensive papillary disease that could not be controlled with transurethral resection and intravesical therapy. ICIC urinary diversion was excluded for patients with distant metastases, severe heart and/or respiratory failure, severe coagulation disorders, severely insufficient renal and/or liver function, or a history of extensive intestinal surgery. Patients were divided into two groups (14 patients in each group) to study the evolution of our surgical technique; the first half group comprised patients who had undergone surgery from January 2018 to July 2019, while the second half group comprised patients who underwent surgery from August 2019 to December 2020. Perioperative variables were analyzed, including patient characteristics, surgical outcome, and perioperative complications. Complications were categorized in accordance with the Clavien–Dindo classification system. Complications of grade 3 or higher were defined as major complications, while those of grade 2 or less were defined as minor complications.

## Surgical technique

### Pre-docking incision

**Fig. 1:**
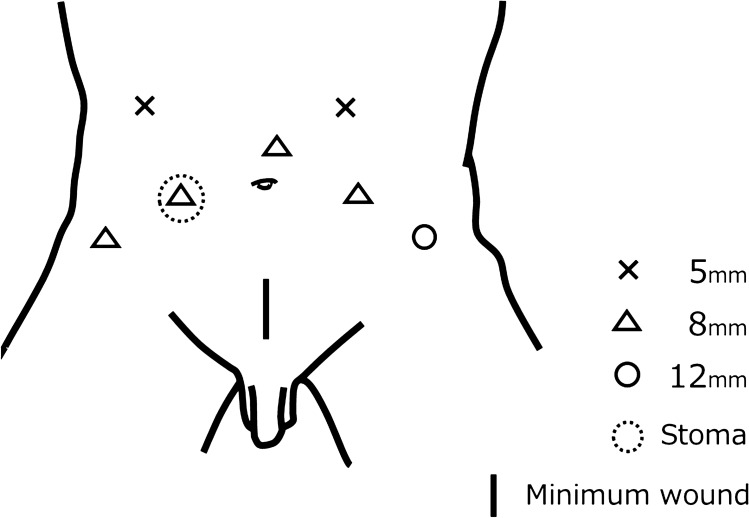
The port locations

All surgical procedures were performed using the da Vinci Surgical System Si or da Vinci Surgical System Xi (Intuitive Surgical, Sunnyvale, CA, USA). Ports were created with the patient in the lithotomy position in the 20-degree Trendelenburg tilt position under general anesthesia. The 20-degree Trendelenburg tilt position moved the intestinal tract to the cranial side so that the intestinal tract did not interfere with port creation. Figure 1 shows the port locations. In addition to creating the ports, a 3.0-cm-long skin incision was made on the cranial side of the pubis and the anterior rectus sheath was incised (minimal wound). Creating a minimal wound at the same time as the port insertion facilitated the removal of the cystoprostatectomy specimen later by the PSS. The stoma was then constructed at its appropriately marked location. The skin and underlying fat were removed at the same time. The stoma was used as the robot port. After the creation of the pre-docking incision, the robot was docked and RARC was performed.

### Removal of the cystoprostatectomy specimen through the minimal wound

After performing RARC and lymph node dissection, the cystoprostatectomy specimen was removed. A tissue storage sack (Anchor II Tissue Retrieval System (bag-alone) 1550 ml; ConMed, Utica, NY, USA) was placed intraperitoneally through the minimal wound by the PSS. The pre-docking incision allowed the PSS to perform the specimen removal procedure seamlessly while the robot was docked. If the procedure was performed without docking, the surgical field could not be maintained due to air leaks when the abdominal cavity was opened. Therefore, we maintained the surgical field and continued the procedure by lifting the abdominal wall with the robot arm during docking. The bag was pulled up gradually using a rotating motion. Excessive pulling force may tear the pouch and lead to inappropriate specimen removal and wound contamination. The Anchor II Tissue Retrieval System, with its excellent durability and sealing ability, prevents bag damage and cancer dissemination, and allows the cystoprostatectomy specimen to be safely removed through the minimal wound.

### Glove port technique

**Fig. 2:**
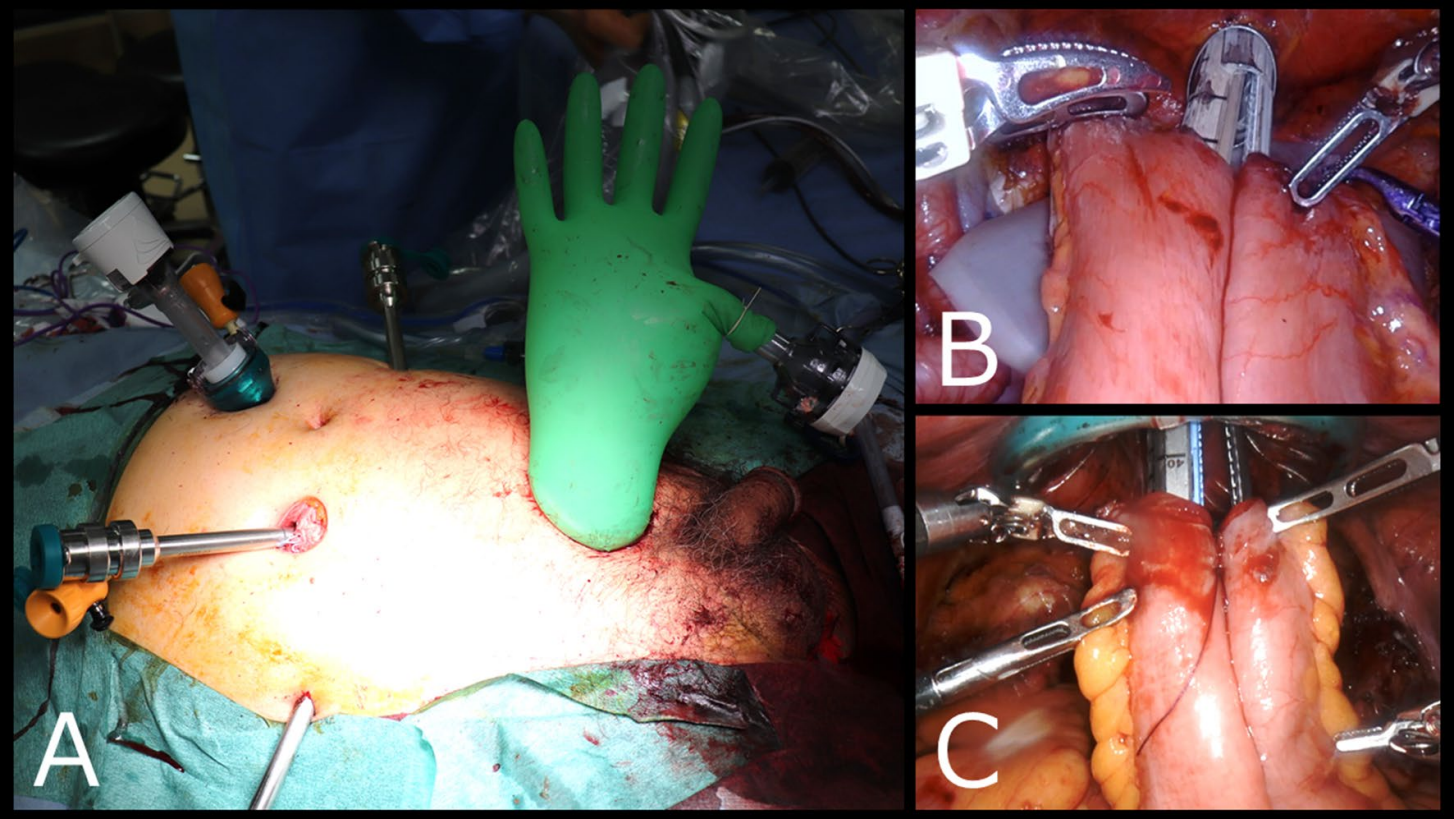
**A** The glove port technique. **B** The patient-side surgeon (PSS) operates an automatic anastomosis device from the normal port. **C** The PSS operates an automatic anastomosis device from the glove port

After the cystoprostatectomy specimen was removed, the minimal wound was used for ICUD using the glove port technique. A retractor (Alexis Wound Retractor XS; Applied Medical, Rancho Santa Margarita, CA, USA) was attached to the minimal wound and used for ICUD as a 12-mm port using surgical gloves (Fig. 2A). At this point, the robot was re-docked with the patient in the 10-degree Trendelenburg tilt position and ICUD was performed. For ileal isolation and anastomosis, the PSS operated an automatic anastomosis device (Powered ECHELON FLEX, suture length 60 mm; Ethicon, Somerville, NJ, USA) through the glove port. Due to the small working space of a normal port, the hinge of the automatic anastomosis device cannot reach the abdominal cavity, making this procedure difficult (Fig. 2B). In contrast, using the glove port widens the working space, making this operation easier (Fig. 2C).

### Passage of the ureteral stents using a laparoscopic suction tip and stay suture technique

**Fig. 3:**
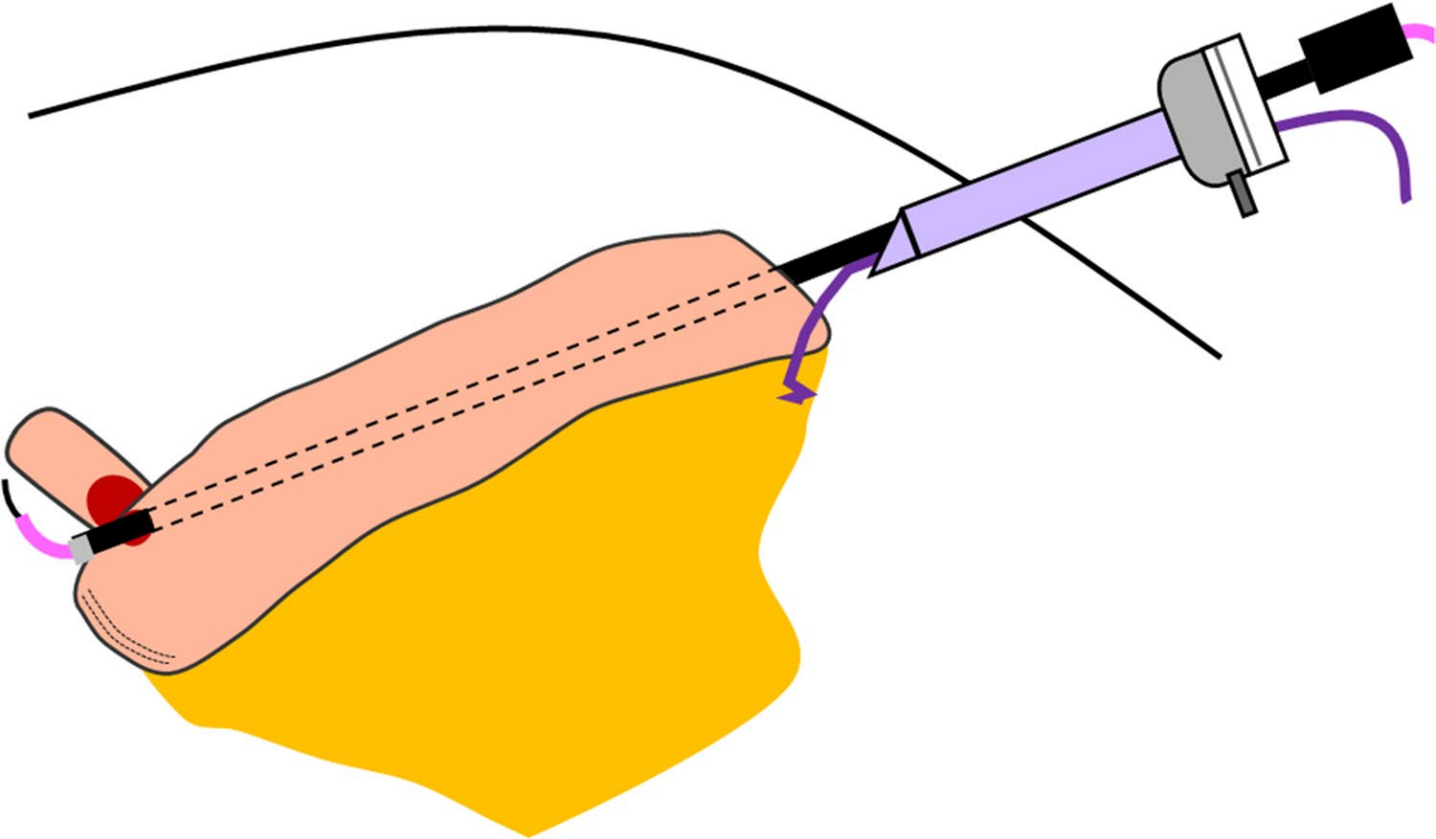
The schema of the passage of the ureteral stents using the laparoscopic suction tip and stay suture technique

After ileal isolation and anastomosis, uretero-ileal anastomosis was performed. Once the posterior wall was anastomosed, ureteral stents were passed into the ureteral end toward the renal pelvis. At our facility, the PSS uses a suction tip and stay suture technique to retrogradely pass a ureteral stent through the ileal conduit (Fig. 3). The mesentery was first ligated on the distal part of the conduit with 3–0 absorbable braided suture (Ethicon 3–0 VICRYL SH), and the suture was guided out of the body through the 5-mm port in the upper right abdomen and used as a stay suture. The suction tip was retrogradely passed through the ileal conduit via the 5-mm port. At this time, the PSS pulled the stay suture and the CS pulled the proximal portion of the conduit to straighten the conduit, facilitating the passage of the suction tip. After the suction tip had passed through the ileal conduit, the PSS advanced the ureteral stent and guidewire through the suction tip, and the CS retrogradely inserted the ureteral stent into the ureteral end toward the kidney.

### Creation of the stoma with the robot docked

**Fig. 4:**
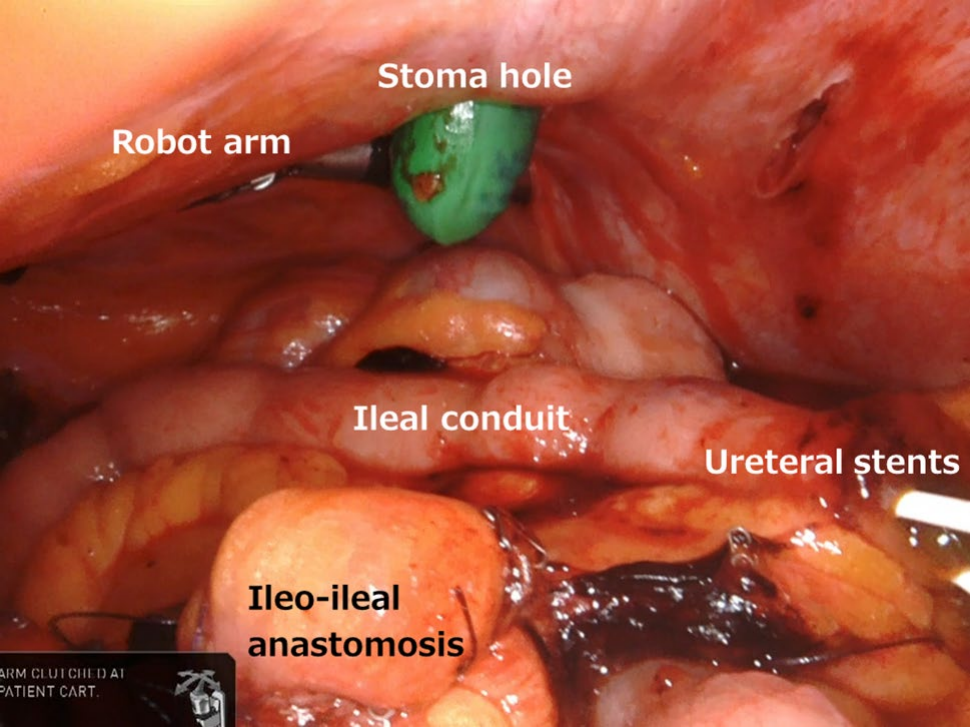
Despite the absence of pneumoperitoneal pressure, the surgical field can be secured by lifting the abdominal wall with the robot arm. The green gloved finger points to the stoma hole

After the robot surgery was completed, a stoma was created. The pre-docking incision allowed the PSS to seamlessly create the stoma. The skin and underlying fat tissue had already been removed, so the surgeon accessed the abdominal cavity simply by incising the fascia. At this time, a stoma was created with the robot still docked. Because the abdominal wall had been lifted by the robot arm, the surgical field was maintained even after the abdominal cavity was opened and the pneumoperitoneum pressure disappeared, so that fascia could be sutured and the distal portion of the ileal conduit was easily pulled up (Fig. 4). To avoid parastomal hernia, the fascia was sutured with 2–0 absorbable braided suture (2–0 VICRYL UR-6; Ethicon, Somerville, NJ, USA) at eight points in advance. After that, the stay suture of the distal part of the conduit was used to pull it through the abdominal wall. The robot was then undocked.

### Statistical analysis

All continuous variables are expressed as the median (interquartile range). The t-test was used to analyze continuous variables, while Fisher's exact test was used for nominal variables. A *p* value of < 0.05 was considered statistically significant. All statistical analyses were performed with EZR (Saitama Medical Center, Jichi Medical University, Saitama, Japan), which is a graphical user interface for R (The R Foundation for Statistical Computing, Vienna, Austria, ver.2.13.0).

## Results

**Fig. 5:**
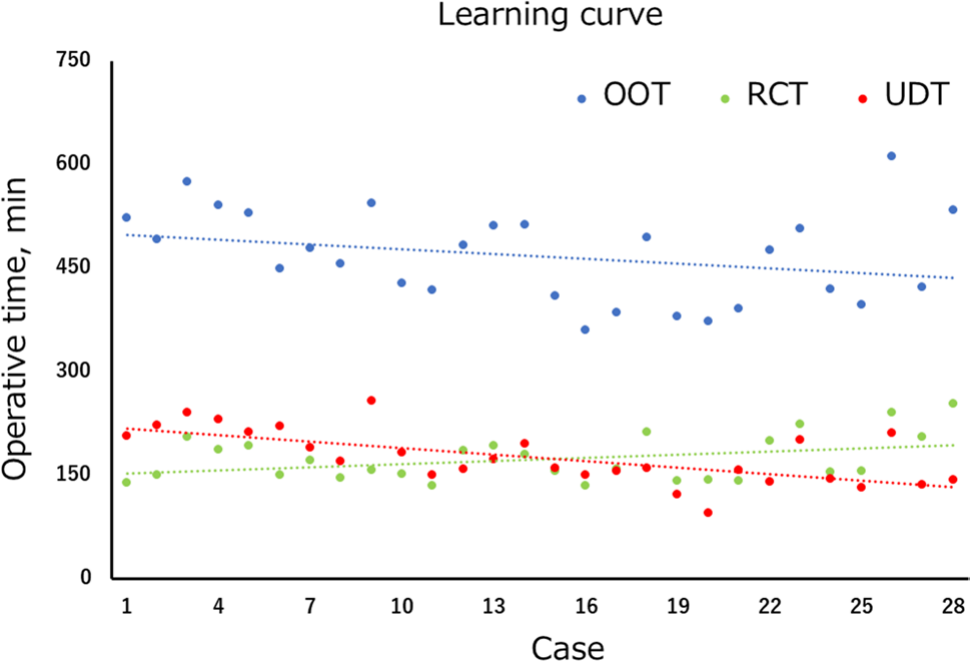
Learning curve

All 28 patients underwent robot-assisted surgery without open conversion. Table 1 shows the patient characteristics and surgical outcomes. The median age was 76 years and the median BMI was 22.9 kg/m^2^. None of the baseline variables significantly differed between the two groups. Bricker anastomosis was significantly more common in the first half group, while Wallace anastomosis was significantly more common in the second half group. The median total surgery time was 478 min, the median RARC time (including pelvic lymph node dissection) was 158 min, and the median ICIC creation time was 165 min. Figure 5 shows the learning curve. The line representing the RARC time was almost flat, but the overall operative time (OOT) and the surgical time for ICIC gradually shortened over time. Compared with the first half group, the second half group had a significantly shorter OOT (501 min vs 414 min, *p* = 0.024) and urinary diversion time (UDT) (202 min vs 148 min, *p* < 0.001). The median specimen removal time, defined as the time from packing the specimen in the tissue storage bag until the tissue was outside the body, was 5.6 min; the specimen removal time tended to be shorter in the second half group than the first half group, but this difference was not significant. Regarding the UDT, the ileal isolation and anastomosis time was significantly shorter in the second half group than the first half group (73 min vs 45 min, *p* < 0.001). In contrast, the uretero-ileal anastomosis time was comparable in the two groups (106 min vs 94 min, *p* = 0.451). Because we thought that Wallace anastomosis would take a shorter time than Bricker anastomosis, we preferentially performed Wallace anastomosis in the second half group. However, changes in the anastomotic technique did not contribute to shortening the UDT. The stenting time was significantly faster in the second half group than the first half group (23.0 min vs 6.5 min, *p* < 0.001). In the first half group, the CS passed the ureteral stents using guidewire. However, this procedure was difficult and took a long time. Therefore, in the second half group, the PSS passed the ureteral stents using a suction tip. In addition, the stay suture allowed the PSS to control the distal portion of the conduit, making the passage of the ureteral stents easier. The median minimal wound length was 3.0 cm. In patients with highly malignant cancer or a large tumor, the minimal wound was extended to prevent dissemination of the cancer due to tissue storage sack damage. The estimated blood loss and transfusion rate did not significantly differ between the two groups.

**Table 1:** Patient characteristics and surgical outcomes

	Overall	First half	Second half	*p* value
	(*n* = 28)	(*n* = 14)	(*n* = 14)	
Age, years, median (IQR)	76 (71–81)	75 (70–79)	76 (71–81)	0.344
BMI, kg/m^2^, median (IQR)	22.9 (20.5–24.5)	23.2 (21.3–25.3)	21.7 (19.9–24.1)	0.154
ASA score ≥ 3, *n* (%)	3 (11)	2 (14)	1 (7)	1.000
Prior abdominal surgery, *n* (%)	8 (29)	4 (29)	4 (29)	1.000
Clinical tumor stage < 2, *n* (%)	4 (14)	2 (14)	2 (14)	1.000
Clinical tumor stage 2 or more, *n* (%)	24 (86)	12 (86)	12 (86)	
Neoadjuvant chemotherapy, *n* (%)	20 (71)	8 (57)	12 (86)	0.077
Pathologic tumor stage < 2, *n* (%)	14 (50)	6 (43)	8 (57)	0.706
Pathologic tumor stage ≥ 2, *n* (%)	14 (50)	8 (57)	6 (43)	
Lymph node yield, median (IQR)	15 (9–21)	12 (8–18)	20 (12–23)	0.324
Positive lymph node, *n* (%)	5 (18)	4 (29)	1 (7)	0.326
Bricker anastomosis, *n* (%)	18 (64)	13 (93)	5 (36)	0.004
Wallace anastomosis, *n* (%)	10 (36)	1 (7)	9 (64)	
OOT, min, median (IQR)	478 (415–515)	501 (462–528)	414 (387–490)	0.024
RCT, min, median (IQR)	158 (149–194)	164 (150–187)	157 (146–211)	0.323
Specimen removal time, min, median (IQR)	5.6 (3.6–10.2)	7.0 (4.5–11.5)	4.9 (3.0–7.7)	0.208
UDT, min, median (IQR)	165 (149–208)	202 (175–222)	148 (137–159)	< 0.001
Ileal isolation and anastomosis, min, median (IQR)	56 (45–71)	73 (69–82)	45 (42–50)	< 0.001
Uretero-ileal anastomosis, min, median (IQR)	101 (81–110)	106 (86–112)	94 (78–105)	0.451
Stenting, min, median (IQR)	11.0 (6.6–22.9)	23.0 (17.6–40.2)	6.5 (6.2–8.3)	< 0.001
Minimum wound length, cm, median (IQR)	3.0 (3.0–3.0)	3.0 (3.0–3.0)	3.0 (3.0–3.0)	0.917
Estimated blood loss, ml, median (IQR)	250 (139–433)	275 (168–424)	231 (108–491)	0.733
Transfusion, *n* (%)	8 (29)	4 (29)	4 (29)	1.000
Time to liquid, POD, median (IQR)	1 (1–3)	1 (1–1)	3 (2–3)	0.022
Time to meal, POD, median (IQR)	4 (3–4)	3 (3–4)	4 (4–5)	0.148
Hospital stay, days, median (IQR)	21 (16–29)	17 (13–26)	25 (21–29)	0.969
30 days’ complication				
Minor complication, *n* (%)	4 (14)	0 (0)	4 (29)	0.098
Major complication, *n* (%)	4 (14)	3 (21)	1 (7)	0.596
90 days’ complication				
Minor complication, *n* (%)	2 (7)	1 (7)	1 (7)	1.000
Major complication, *n* (%)	0 (0)	0 (0)	0 (0)	1.000

*BMI* body mass index, *ASA* American society of anesthesia, *OOT* overall operative time, *RCT* radical cystectomy time, *UDT* urinary diversion time, *POD* postoperative day

The incidence of complications of all grades within the first 30 days postoperatively was 28.6%. Overall, four patients (14%) had major complications of Clavien–Dindo grade 3 or higher. Three patients in the first half group had major complications, including an infected lymphocele that required percutaneous drainage in two patients (Clavien–Dindo grades 3a and 3b), and a conduit-enteric fistula requiring fistula closure surgery in one patient (Clavien–Dindo grade 3b). In the second half group, four patients had minor complications (Clavien–Dindo grade 2 UTI treated with antibiotics in two patients, and ileus treated conservatively in two patients) and one had a major complication (panperitonitis due to rectal injury that required a temporary colostomy). The incidence of complications of all grades 30–90 days postoperatively was 7.1%; one patient in each of the two groups developed UTI that was treated with antibiotics (Clavien–Dindo grade 2). No major complications were observed 30–90 days after surgery, and all major complications occurred within 30 days after surgery. No complications occurred due to the PSS surgical techniques.

## Discussion

Since its introduction at the beginning of the twenty-first century, the da Vinci surgical robot has revolutionized laparoscopic surgery, especially urologic and pelvic surgery. However, the surgical robot has shifted the main surgeon away from the patient’s side so that they no longer control the patient directly on the operating table. As a result, robotic surgery mandates the presence of another surgeon who is scrubbed in and stands as an assistant on the patient's side. This assistant must be well-skilled in technical work associated with the robotic patient-side cart and provide pure laparoscopic assistance. This makes the role of the PSS even more important in robotic surgery than in pure laparoscopic surgery [2]. A trained PSS who can skillfully accomplish his/her role during the procedure is considered essential for the establishment of a successful robotic program [3]. In urologic surgery, the PSS plays a key role in ICUD. Among the ICUD procedures, the creation of the ICIC was the subject of our research. The surgical techniques performed by the PSS in the creation of the ICIC and intracorporeal neobladder include the removal of the specimen through the minimal wound and the glove port techniques. In addition, the PSS performs more surgical techniques for the creation of the ICIC than the intracorporeal neobladder, such as the passage of the ureteral stent and creation of the stoma.

Studies evaluating ICUD have focused on its feasibility and surgical outcomes, and the experience or learning curve of the CS [4, 5]. However, there has been little discussion about when, where, and how to remove the specimen in RARC with ICUD. One of the challenges of laparoscopic surgery is how to retrieve the specimen after excision with a minimal wound. Only the port wounds are required for laparoscopic surgery without specimen removal and for laparoscopic surgery with the removal of benign tissue, as the resected specimen can be morcellated in the abdominal cavity. However, laparoscopic surgery for malignant diseases requires en bloc specimen removal because it is unacceptable to morcellate the specimen in the abdominal cavity due to the risk of cancer dissemination and the need for accurate histopathological evaluation. Minimally invasive laparoscopic surgery for malignant diseases refers to surgery performed with the smallest possible wound required to remove the specimen. Thus, MIS for RARC in men is surgery with the smallest possible wound required to remove the cystoprostatectomy specimen. Women undergoing RARC were excluded from this study because the specimen is removed through the vagina. If a large skin incision is made when removing the cystoprostatectomy specimen, RARC with ICUD may not be MIS. Furthermore, some urologists argue that extracorporeal urinary diversion, not ICUD, should be performed through a large wound. The PSS performs the surgical techniques required to remove the bladder from the body. We decided to remove the specimen before ICUD through the minimal wound, which was subsequently used for ileal isolation and anastomosis with the glove port technique. Specimens should be removed prior to ICUD to reduce the risk of cancer dissemination as much as possible. The pre-docking incision made the specimen removal seamless. The glove port technique widened the working space of the PSS, which facilitated the ileal isolation and anastomosis with an automatic anastomosis device and resulted in a shorter time for this surgical step. During the specimen removal and stoma creation, pneumoperitoneum pressure was lost by opening the abdominal cavity. However, lifting the abdominal wall with a robot arm maintained the surgical field and facilitated the PSS procedure.

The passage of the ureteral stents in ICUD can be challenging compared with extracorporeal urinary diversion. Coordination between the PSS and the CS is vital [6]. Various methods are used for the passage of the ureteral stents in ICUD. Robot forceps are directly inserted in the ileal conduit when the CS operates. When the PSS operates, various instruments, such as intestinal grasping forceps and a suction tip, are used. When using a guidewire, the procedure depends on whether the CS or the PSS operates and whether the ureteral stent is passed through the ileum in an antegrade or retrograde manner. In addition, the difficulty varies depending on whether the uretero-ileal anastomosis is done using the Bricker or the Wallace technique and on the length of the conduit. To minimize damage to the ileal conduit, the PSS uses a suction tip and stay suture technique. The blunt suction tip is gently guided through the ileal conduit [7], and the stay suture allows the PSS to control the distal portion of the conduit, making the passage of the ureteral stents easier and resulting in a significantly shorter stenting time.

The present study had some limitations. First, it was a retrospective study with a small sample size. Second, it was difficult to quantitatively evaluate the surgical technique of the PSS. In this study, a quantitative evaluation was made by comparing the time required for each step of the operation. However, the surgical time may not have reflected only the PSS techniques, as many factors affect the surgery time. Future studies should assess other objective data that evaluate the PSS techniques, such as CS satisfaction questionnaire data.

## Conclusion

We described the surgical techniques of the PSS in the creation of the ICIC and showed that these techniques facilitated the creation of the ICIC. As the experience of the PSS increased, the surgical time of procedures involving the PSS tended to decrease.
